# Investigating Roles of Cerebral Blood Flow to Maintain Thermal Stability of Neonatal Brain Against Cold Stress Using Non-Invasive Probes for Brain Perfusion and Temperature Gradient

**DOI:** 10.3390/bios16020127

**Published:** 2026-02-20

**Authors:** Sachiko Iwata, Kennosuke Tsuda, Masahiro Kinoshita, Shinji Saitoh, Osuke Iwata

**Affiliations:** 1 Center for Human Development and Family Science, Department of Pediatrics and Neonatology, Nagoya City University Graduate School of Medical Sciences, Nagoya 467-8601, Japan; kentsuda@med.nagoya-cu.ac.jp (K.T.); ss11@med.nagoya-cu.ac.jp (S.S.); 2 Centre for Developmental and Cognitive Neuroscience, Department of Paediatrics, Kurume University School of Medicine, Kurume 830-0011, Japan; kinoshita_masahiro@kurume-u.ac.jp

**Keywords:** ambient temperature, brain temperature, cerebral blood flow, cold stress, preterm infant

## Abstract

Background: Brain temperature is an important determinant of neurological outcomes in ill infants, yet contributions of environmental temperature and cerebral blood flow remain uncovered because of the lack of non-invasive probes. Methods: Using non-invasive cot-side probes, we examined how cerebral blood flow influences brain temperature during mild cold stress induced by incubator-to-cot transfer. We studied 43 clinically stable infants in a tertiary neonatal intensive care unit. After cot transfer, infants were routinely fitted with knit caps and wrapped in cotton blankets. Scalp and superficial and deep brain temperatures were measured using infrared and zero-heat-flux thermometers, and superior vena cava (SVC) flow—a proxy for cerebral blood flow—was assessed using Doppler velocimetry before, immediately after, and 2 h after transfer, adjusting for rectal temperature. Results: Ambient temperature decreased from 29.7 (SD 0.8) °C to 26.8 (SD 0.9) °C, while rectal temperature remained stable. Scalp and brain temperatures declined after transfer but superficial and deep brain temperatures returned to baseline after 2 h of cap use. The regression coefficient between SVC flow and superficial brain temperature shifted from −0.176 (95% CI, −0.386 to 0.035) to 0.239 (−0.280 to 0.759) after transfer (difference: 0.415 [0.106 to 0.724]; *p* = 0.009), and then returned to baseline after 2 h (−0.079 [−0.528 to 0.372]). Conclusions: Relationships between brain temperature and perfusion were successfully monitored using non-invasive cot-side biosensors; cerebral blood flow appears to shift from facilitating heat dissipation in warm conditions to supporting heat delivery during cold stress. These findings underscore the physiological role of cerebral blood flow in maintaining brain temperature.

## 1. Introduction

Preterm infants are vulnerable to hypothermia due to excessive heat dissipation from immature skin and a limited capacity for heat production [1,2]. Postnatal hypothermia impairs short-term cardiorespiratory transition and affects long-term developmental outcomes [3,4]. Knit caps, radiant heaters, temperature-controlled incubators, and, more recently, plastic bags have been used to prevent hypothermia following preterm birth [5]. These studies consistently monitored axillary and rectal temperatures as proxies for core body temperature. Although the brain is a central site for heat production and dissipation [6,7], the brain temperature distribution and its regulation in preterm infants remain poorly understood, because of the difficulty in non-invasively assessing the cerebral blood flow and brain temperature in vulnerable infants.

Although direct monitoring of brain temperature and perfusion is clinically challenging, estimating cerebral blood flow, oxygen metabolism, and temperature is feasible using multimodal non-invasive biosensors, such as time-resolved near-infrared spectroscopy, zero-heat-flux thermometry, infrared thermometry, and Doppler ultrasound. Although less widely used, zero-heat-flux thermometry non-invasively estimates deep tissue temperature by eliminating heat loss at the skin surface using active thermal compensation; once thermal equilibrium is achieved, the measured surface temperature theoretically reflects that of the underlying tissue [8,9]. By integrating these non-invasive bedside probes, our group has developed an original platform for the assessment of cerebral blood flow, oxygen metabolism, and thermal regulation. These studies revealed that, in preterm infants managed in closed incubators, higher cerebral blood flow was associated with lower superficial brain temperatures and a larger intracranial temperature gradient, suggesting that cerebral perfusion facilitates heat removal from the tissue in warmer environments [10]. Conversely, in a separate cohort managed in open cots, higher cerebral blood flow was associated with higher superficial brain temperatures and a smaller intracranial temperature gradient, suggesting that cerebral perfusion delivers heat to the tissue in cooler environments [11]. While these cross-sectional findings suggest that cerebral blood flow is critical for maintaining thermal stability, this hypothesis requires validation in a longitudinal study that serially monitors changes in flow–temperature relationship under varying ambient temperatures.

A prospective observational study was conducted in clinically stable hospitalized infants to investigate the relationship between cerebral blood flow and brain temperature and how it depends on the temporal change in ambient temperature caused by closed-to-open cot transfer, by combining non-invasive bedside probes.

## 2. Materials and Methods

### 2.1. Participants

A total of 43 infants managed in closed incubators (Dual IncuI; Atom Medical, Tokyo, Japan) at a tertiary neonatal intensive care centre of Kurume University Hospital were recruited. Participants had been weaned from intensive cardiorespiratory support and were scheduled for transfer from a closed to an open cot. In this unit, open cot transfer is considered for infants weighing > 1600 g whose rectal temperature remains >36.5 °C at an incubator temperature setting of ≤31 °C. Following transfer, infants were dressed in knit cap and socks and covered with a blanket to prevent hypothermia. Return to the closed incubator is considered if body temperature fails to remain ≥36.5 °C.

### 2.2. Data Collection

Data on gestational age, body weight, postnatal age, and head circumference at the time of the study were obtained from the electronic medical records.

Temperature measurements and echocardiographic examinations were conducted at three time points: approximately 30 min before transfer, 10 min after transfer (before knit cap application), and 2 h after transfer. Data were collected following previously established protocols [10,11]. Briefly, assessments were performed while the infants were asleep or quietly awake approximately 1 h after feeding. All the data were collected by the same research team in a standardized sequence to minimize technical bias. S.I. performed echocardiographic examinations, while K.T. and O.I. performed temperature measurements. All procedures were completed within 20 min.

Scalp temperature (T_scalp_) was measured at the centre of the forehead using a non-contact infrared thermometer (Thermofocus Pro; Technimed, Varese, Italy). Superficial and deep brain temperatures were assessed using a zero-heat-flux thermometer (Coretemp; Terumo, Tokyo, Japan) and two probes of different diameters [12]. Because probe diameter theoretically corresponds to tissue measurement depth, a 25 mm probe was applied to the anterior fontanelle to estimate deep brain temperature (T_deep brain_), whereas a 15 mm probe was placed on the forehead to estimate superficial brain temperature (T_superficial brain_). The rectal temperature (T_rectal_) was measured at a depth of 3 cm from the anal margin using a thermistor probe (C202; Terumo, Tokyo, Japan). The ambient temperature and humidity in the closed incubator and open cot were measured using a thermohygrometer (605-H1 Mini; Testo, Yokohama, Japan) approximately 10 cm above the infant’s face. Because regional body temperatures depend on core temperature, scalp and brain temperatures were adjusted for T_rectal_ to yield relative values (rT_scalp_, rT_superficial brain_, and rT_deep brain_).

Echocardiographic measurements were performed by an experienced neonatologist (S.I.) using a high-frequency (8–13 MHz) vector array transducer (iE33; Philips, Amsterdam, The Netherlands). Superior vena cava (SVC) was visualised from a low subcostal view by angling the probe anteriorly to identify flow entering the right atrium using colour Doppler. The velocity–time integral (VTI) was calculated from 10 consecutive cycles. The SVC diameter was measured at the junction with the right atrium, from frozen images of the vessel walls in systole and diastole. The mean SVC diameter was derived from 3 to 5 cardiac cycles. SVC flow was then calculated using the formula [13]:

SVC flow = V_SVC_ × HR × π × (D_svc_)^2^/4

where

V_svc_ = SVC velocity–time integral (cm);

HR = heart rate (beats/min);

D_svc_ = mean SVC diameter (cm).

To account for cerebral relevance, SVC flow was normalised to 100 g of brain weight (rSVC), with brain mass estimated from head circumference [14]. This method and its clinical utility as a proxy for cerebral perfusion have been validated in preterm infants [13,15].

### 2.3. Data Analysis

Values are expressed as frequency (%), mean (standard deviation), or mean (95% confidence interval), unless otherwise specified. A mixed effects model was employed to account for repeated measurements within individuals, with patient identity included as a random effect and measurement timing as a fixed effect. To examine whether the relationship between rSVC flow and scalp and brain temperatures differed before and after cot transfer, interaction terms were included in the model. Continuous independent variables were mean-centered to facilitate interpretation.

Statistical analyses were performed using IBM SPSS Statistics version 24 (IBM Japan, Tokyo, Japan). Statistical significance was set at *p* < 0.05.

## 3. Results

The reasons for hospitalization were preterm birth and/or low birth weight (n = 19), hypoglycaemia (n = 11), respiratory failure (n = 7), maternal autoimmune disease (n = 3), and feeding difficulties (n = 3). None of the infants had major congenital anomalies or brain lesions.

**Figure 1:**
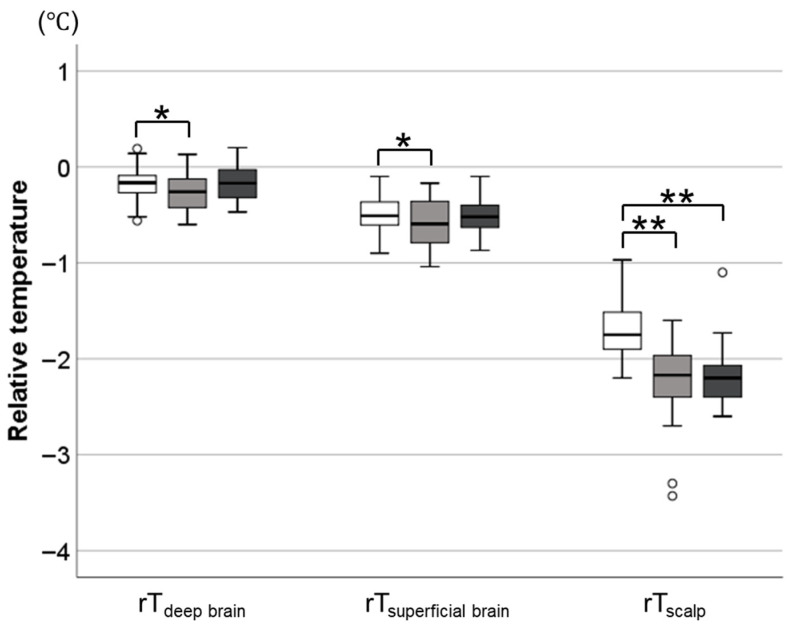
Temporal Changes in Relative Temperatures of Brain and Scalp in Response to Cot Transfer. Box plots indicating body temperatures during closed incubator management (white box), immediately after cot transfer (grey box), and 2 h after transfer (black box). * *p* < 0.05 and ** *p* < 0.001 vs. incubator management. rT_scalp_ showed a significant drop after cot transfer and remained low for 2 h, whereas rT_deep brain_ and rT_superficial brain_ showed only a transient decrease and returned to baseline within 2 h. Abbreviations: T_rectal_, rectal temperatures. rT_deep brain_, rT_superficial brain_ and rT_scalp_, relative brain and scalp temperatures to rectal temperature.

Ambient humidity did not differ significantly across the three time points, whereas ambient temperature decreased from 29.7 (0.8) °C to 26.8 (0.9) °C after cot transfer and remained depressed until 2 h after transfer (Table 1). In contrast to rT_scalp_, which decreased significantly after transfer and remained depressed for 2 h, rT_deep_ brain and rT_superficial brain_ decreased only transiently after transfer, returning to baseline level after 2 h (shown in Figure 1).

**Figure 2:**
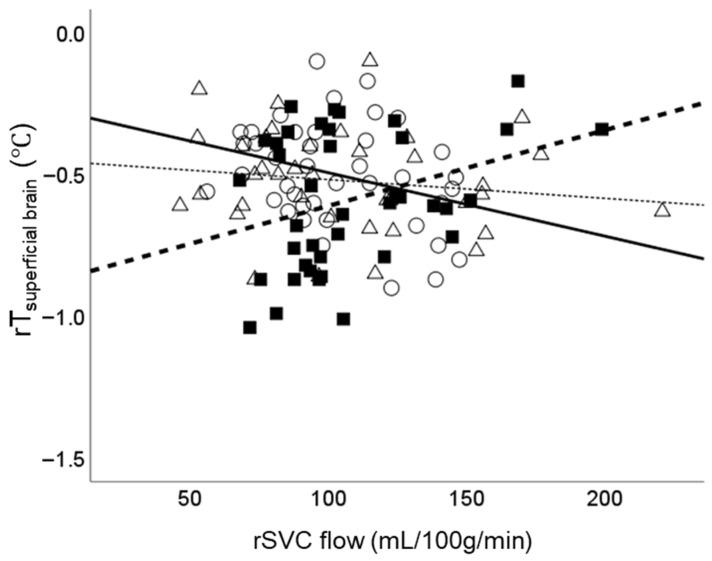
Relationship between Cerebral Blood Flow and Relative Superficial Brain Temperature. Scatter plot showing a negative relationship between rT_superficial brain_ and rSVC during the closed incubator care (open circle with black solid regression lines), which changed to a positive relationship immediately after the cot transfer (black square with dashed regression lines). However, 2 h after cot-transfer (open triangle with dotted regression lines), the relationship returned to a pattern similar to the closed incubator care. Abbreviation: rT_superficial brain_, relative superficial brain temperature to rectal temperature.

The associations between temperature variables, timing, and rSVC flow, along with their respective interactions, were analysed using multivariable models (Table 2). Cot transfer was associated with lower rT_deep brain_ and rT_superficial brain_ (*p* = 0.030 and 0.043, respectively); these associations were no longer observed after 2 h (Table 2). In contrast, cot transfer was associated with lower rT_scalp_ immediately after cot transfer; this association persisted until 2 h after cot transfer (both *p* < 0.001). With regard to their interactions, cot transfer was associated with a temporal shift in the relationship between rSVC flow and rT_superficial brain_; the regression coefficient for the relationship between rSVC flow and rT_superficial brain_ shifted from −0.176 (95% confidence interval [CI], −0.386 to 0.035) before transfer to 0.239 (95% CI, −0.280 to 0.759) immediately after transfer (difference: 0.415 [0.106 to 0.724]; *p* = 0.009) (Table 2). However, after 2 h of cot transfer, the regression coefficient returned to −0.079 (−0.528 to 0.372) (difference: 0.097 [−0.142 to 0.337]; *p* = 0.418) (Table 2). A scatter plot of the raw data corresponding to the multivariable model in Table 2 is presented in Figure 2 for visual inspection, illustrating the temporal shift in the relationship between rSVC and rT_superficial brain_.

## 4. Discussion

Despite the critical role of cerebral thermal regulation in determining the outcomes of preterm infants, the mechanisms involved remain poorly understood due to a lack of non-invasive probes for measuring cerebral blood flow and brain temperature. By integrating multimodal non-invasive sensors—specifically zero-heat-flux thermometry, infrared thermometry, and Doppler ultrasound—this study successfully characterised the physiological responses involved in maintaining cerebral thermal stability in clinically stable, hospitalised preterm infants, who were exposed to mild cold stress after open-cot transfer. The reduction in ambient temperature induced a significant decrease in scalp and superficial brain temperatures, resulting in a temporary reversal in the relationship between brain perfusion and temperature. These findings support the hypothesis derived from our previous cross-sectional studies that cerebral blood flow shifts from mediating heat dissipation to facilitating heat delivery in cold environments. After thermal equilibrium was achieved using knit caps, the perfusion–temperature relationship returned to pre-transfer baseline levels; this suggests that knit caps provide thermal insulation comparable to the warm ambient environment of a closed incubator. Further studies on brain temperatures in high-risk infants are required to confirm the role of cerebral blood flow as an active regulator of regional brain temperatures.

Recent technological developments in cerebral monitoring have facilitated the assessment of brain temperature, perfusion, metabolism, and function without the insertion of invasive probes into the brain tissue. For example, three-dimensional brain temperature estimation using magnetic resonance spectroscopy [16] and MRI-based perfusion imaging [17] have already been introduced into clinical practice of adult patients. However, transporting fragile neonates to MRI suites presents significant challenges, given the risks of physiological instability and their high susceptibility to hypothermia during the procedure. Moreover, the requirement to perform assessments outside the neonatal intensive care unit makes it exceedingly difficult to monitor real-time physiological responses to clinical interventions and environmental changes. These limitations have hindered progress in elucidating these regulatory mechanisms, despite the critical importance of brain temperature and its confounders for the neurological outcomes of preterm neonates.

To move beyond these methodological limitations, our group has integrated multimodal bedside biosensors to develop an experimental setup for the non-invasive assessment of regional brain temperature, perfusion, and oxygen metabolism of the neonatal brain within the intensive care setting [10,11]. To assess brain tissue temperature, we employed zero-heat-flux core temperature monitoring, which utilises a heater and a thermal flux transducer to establish thermal equilibrium between superficial and deep tissue structures [8,9]. The validity of the same system with ours was established by Matsukawa et al., showing that a 43 mm diameter sensor element can monitor temperatures at depths of 18–38 mm, effectively reflecting deep tissue thermal states [12]. Since smaller probes were used for the current study cohort, the measurement depth was postulated to reach superficial brain tissues at approximately 10–20 mm from the surface according to their diameters. SVC flow was measured using Doppler ultrasonography following an established method that multiplies the SVC flow velocity by the mean vessel diameter [13,15]. Since Kluckow and Evans first reported this technique, a number of researchers have adopted it as a proxy for cerebral perfusion and reported its relevance to clinical outcomes, such as the incidence of intraventricular haemorrhage, mortality, and neurodevelopmental outcomes [18]. By integrating multimodal non-invasive sensors, this study successfully characterised the physiological responses involved in maintaining cerebral thermal stability in clinically stable, hospitalised preterm infants, who were exposed to mild cold stress after open-cot transfer.

In our previous cross-sectional studies, higher cerebral blood flow was associated with lower brain temperature in infants managed in closed incubators, suggesting that blood flow facilitates heat dissipation in warm environments [10]. In contrast, in a cohort of hospitalised infants managed in open cots, higher cerebral blood flow was paradoxically associated with higher brain temperature, suggesting that blood flow serves to deliver heat to the brain in cool environments [11]. The current longitudinal study confirmed that the reduction in brain temperature after transfer shifted the perfusion–temperature relationship from negative to positive. Unlike our previous study, which assessed infants prior to the scheduling of transfer, the current study was conducted specifically on the day of transfer. Likely because the ambient temperature within the incubator had already been lowered in preparation for weaning, the baseline perfusion–temperature relationship did not reach statistical significance. However, following transfer, the regression coefficient changed significantly from negative to positive, consistent with a transition from heat dissipation to delivery. Cerebral blood flow is primarily responsible for the delivery of oxygen and energy substrates [19,20]. However, our data reinforce the concept that cerebral blood flow actively modulates brain temperature in response to environmental stimuli, potentially serving as an integral component of thermal and metabolic homeostasis [7,21].

In immature brain tissue, the supply–demand balance of oxygen and energy substrates is fragile and easily disrupted by stressful stimuli occurring during labour and birth transition [22]. Infants are inevitably exposed to cold environments immediately after delivery. Common clinical conditions such as low birth weight and prolonged resuscitation may induce significant temperature fluctuations after birth [1,2]. The impact of environmental heat exchange is pronounced in the immature brain, because the fraction of heat dissipation and production via the cranial region is significantly greater in infants than in adults [23]. Indeed, our current data suggest that even when normal rectal temperature is maintained after cot transfer, scalp and superficial brain temperatures decrease significantly, resulting in an increased intracranial temperature gradient. Given that tissue oxygen metabolism is temperature-dependent [24,25], even local temperature changes may substantially alter tissue oxygen demand. Therefore, without efficient vascular autoregulation, the balance between tissue perfusion and metabolism may be disrupted. Taken together, the presence of temperature-guided regulation of regional brain perfusion is particularly relevant in infants and warrants confirmation in future studies.

The mechanisms underlying temperature-guided regulation of cerebral blood flow remain incompletely understood. Among key mediators, oxygen and carbon dioxide partial pressures play opposing roles in regulating cerebrovascular tone; a decrease in arterial oxygen tension induces local vasodilation via hypoxic mechanisms to preserve oxygen delivery [26], whereas a reduction in carbon dioxide tension—common during hypothermia due to reduced metabolic activity—leads to vasoconstriction [27]. In our cohort, scalp and superficial brain temperatures declined after cot transfer despite stable rectal temperature, indicating the presence of localised cold stress in superficial regions. Although not monitored in our study, it is plausible that regional temperature reductions altered local oxygen and carbon dioxide tensions via a leftward shift in the haemoglobin-oxygen dissociation curve [23]. Reduced partial oxygen tension in the local tissue may trigger compensatory vascular responses to maintain the flow–metabolism coupling [23,24,25], where regional perfusion is adjusted to meet oxygen demand [28,29]. Contrary, temperature-related hypocapnia may have simultaneously counteracted this response by inducing vasoconstriction, resulting in complex net effects. These opposing influences—hypoxia-induced vasodilation and hypocapnia-induced vasoconstriction—may need to be considered in understanding the observed phenomenon. In our study, the regression coefficient between rSVC flow and superficial brain temperature shifted from negative to positive, suggesting that hypoxia-induced vasodilation was predominant over other counteracting factors. Such a complex regulatory mechanism may explain the relatively modest changes in perfusion observed in this study. Further studies incorporating simultaneous gas measurements are needed to clarify the dynamic interplay between cerebral temperature, oxygen/carbon dioxide tension, and vascular tone in the neonatal brain.

In the current study, no significant reduction in T_rectal_ after cot transfer was noted; therefore, no infant required return to the closed incubator, indicating that the current measures to prevent hypothermia after cot transfer are effective. Despite the maintenance of normal T_rectal_, scalp and superficial brain temperatures dropped significantly after transfer; however, these values recovered after 2 h of knit cap use. Immediately after cot transfer, a higher ambient temperature was significantly associated with higher rT_scalp_. However, after 2 h of wearing a knit cap, the regression slope between ambient temperature and rT_scalp_ became nearly zero, closely resembling the slope observed during incubator care before transfer, although the absolute rT_scalp_ remained approximately 0.7 °C lower (Online Supplementary Figure S1). This suggests that the knit cap was as effective as the warm ambient environment of a closed incubator in maintaining thermal stability. Knit caps have been empirically used in hospitalised infants as insulators to minimise heat loss from the scalp [5]. The current findings support the benefit of using knit caps after cot transfer to preserve cerebral thermal stability. However, it is also possible that thermal equilibrium after 2 h of cot transfer, rather than the knit cap itself, contributed to the thermal stability of the infant brain, as no infant in the current study cohort was managed without a knit cap after the transfer. Future studies should assess the role of thermal insulators, such as knit caps, in replicating the thermal environment of a closed incubator.

Our study was conducted in clinically stable, hospitalised infants who had transitioned beyond the acute phase. Thus, we were able to elucidate aspects of the physiological mechanism of cerebrovascular autoregulation in response to temperature changes in relatively healthy infants. Our current findings suggest the presence of active brain temperature regulation via cerebral blood flow. However, these findings may not apply to critically ill or extremely preterm infants dependent on intensive care, whose cerebrovascular autoregulation may be compromised. For ethical and safety reasons, we did not alter the ambient temperature; instead, we collected serial data before and after the inevitable temperature drop associated with cot transfer. Therefore, the observed ambient temperature drop was small, leading to limited changes in brain temperature and perfusion. Given the limited sample size, the small magnitude of observed changes, and the multiple comparisons conducted across different temperature measurement locations, these associations should be interpreted with caution. We monitored the SVC flow velocimetry to estimate brain perfusion. However, we were unable to assess spatial heterogeneity in brain perfusion. In addition, SVC flow in infants is known to include 20–30% of blood flow from extracerebral tissues [13]. Further studies are warranted to investigate the vascular response to cold stress in immature infants.

## 5. Conclusions

By integrating multimodal non-invasive sensors—specifically zero-heat-flux thermometry, infrared thermometry, and Doppler ultrasound—this study successfully characterised the physiological responses involved in maintaining cerebral thermal stability in clinically stable, hospitalised preterm infants, who were exposed to mild cold stress after open-cot transfer. Consistent with findings from previous cross-sectional studies, mild cold stress after cot transfer and the subsequent reduction in superficial brain temperature shifted the perfusion–temperature relationship from heat removal to heat delivery. These results support the hypothesis that cerebral blood flow is crucial for regulating brain temperature, in addition to delivering oxygen and energy substrates. Given the significant temporal changes in cerebral perfusion after birth and inter-individual variations related to maturity and disease, greater attention needs to be paid to brain temperature to ensure that cerebral metabolic demands are met. In relatively mature infants, the use of a knit cap in cooler environments may partially compensate for the reduction in ambient temperature after transfer from a closed incubator.

## Figures and Tables

**Table 1:** Clinical Background and Physiological Variables before and after Cot Transfer.

	Before	Post 0 h	Post 2 h
Clinical background variables			
Female sex	18		
Gestational age (week)	37.0 (3.2)		
Post-natal age (d)	7 (14)		
Body weight at cot transfer (g)	2567 (512)		
Head circumference at cot transfer (cm)	32.7 (2.0)		
Ambient condition			
Temperature (°C)	29.7 (0.8)	26.8 (0.9) **	26.9 (0.8) **
Humidity (%)	49.0 (9.4)	50.3 (12.8)	49.1 (14.1)
Temperature measures (°C)			
T_rectal_	37.0 (0.2)	37.1 (0.3)	37.0 (0.2)
T_deep brain_	36.8 (0.0)	36.8 (0.3)	36.8 (0.2)
T_superficial brain_	36.5 (0.2)	36.5 (0.3)	36.5 (0.2)
T_scalp_	35.3 (0.3)	34.9 (0.5) **	34.8 (0.3) **
rT_deep brain_	−0.18 (0.16)	−0.26 (0.18) *	−0.18 (0.18)
rT_superficial brain_	−0.50 (0.18)	−0.59 (0.24) *	−0.52 (0.18)
rT_scalp_	−1.72 (0.28)	−2.20 (0.40) **	−2.20 (0.28) **
Physiological variables			
Heart rate per minute	128 (13)	130 (16)	131 (13)
Oxygen saturation (%)	99 (0.9)	99 (0.9)	99 (1.3)
rSVC flow (mL/100 g/min)	103.3 (25.2)	107.7 (29.0)	106.5 (40.0)

Values are presented as mean ± SD. * *p* < 0.05 and ** *p* < 0.001 vs. before transfer. Abbreviations: T_rectal_, rectal temperatures. T_deep brain_ and T_superficial brain_, brain temperatures, measured using a zero-heat flux thermometer with probe diameters of 25 mm and 15 mm. T_scalp_, scalp temperature at the forehead. rT_deep brain_, rT_superficial brain_ and rT_scalp_, relative brain and scalp temperatures to rectal temperature. rSVC, the relative blood flow of the superior vena cava.

**Table 2:** Dependence of Body Temperatures on Ambient Settings, Cerebral Blood Flow and Their Interactions.

Variables		T_rectal_				rT_deep brain_				rT_superficial brain_				rT_scalp_			
		Regression coefficient			*p*	Regression coefficient			*p*	Regression coefficient			*p*	Regression coefficient			*p*
		Mean	95% CI			Mean	95% CI			Mean	95% CI			Mean	95% CI		
			Lower	Upper			Lower	Upper			Lower	Upper			Lower	Upper	
Timing	Before	Reference				Reference				Reference				Reference			
	Post 0 h	0.041	−0.067	0.150	0.447	−0.076	−0.145	−0.008	**0.030**	−0.084	−0.165	−0.003	**0.043**	−0.490	−0.627	−0.354	**<0.001**
	Post 2 h	−0.040	−0.125	0.046	0.355	0.013	−0.052	0.077	0.696	−0.017	−0.085	0.052	0.623	−0.487	−0.605	−0.369	**<0.001**
rSVC (×10^−2^) (mL/100g/min)		−0.039	−0.320	0.243	0.782	−0.129	−0.318	0.059	0.173	−0.176	−0.386	0.035	0.099	0.098	−0.266	0.462	0.589
Interaction	Before × rSVC	Reference				Reference				Reference				Reference			
	0 h × rSVC (×10^−2^)	−0.038	−0.442	0.367	0.853	0.105	−0.158	0.368	0.428	0.415	0.106	0.724	**0.009**	0.236	−0.282	0.754	0.366
	2 h × rSVC (×10^−2^)	0.030	−0.276	0.337	0.843	0.007	−0.214	0.228	0.949	0.097	−0.142	0.337	0.418	−0.269	−0.682	0.144	0.198

Abbreviations: T_rectal_, rectal temperatures. T_deep brain_, rT_superficial brain_ and rT_scalp_, relative brain and scalp temperatures to rectal temperature. rSVC, the relative blood flow of the superior vena cava. CI, confidence interval. *p*-values < 0.05 are indicated in **bold**.

## Data Availability

Data presented in this study are available upon request from the corresponding authors.

## Supplementary Materials

The following supporting information can be downloaded at: https://www.mdpi.com/article/10.3390/bios16020127/s1, Figure S1: Relationship between Ambient Temperature and Relative Scalp Temperature.

## References

[1] Beletew B., Mengesha A., Wudu M., Abate M. (2020). Prevalence of neonatal hypothermia and its associated factors in East Africa: A systematic review and meta-analysis. BMC Pediatr..

[2] Knobel R., Holditch-Davis D. (2007). Thermoregulation and heat loss prevention after birth and during neonatal intensive-care unit stabilization of extremely low-birthweight infants. J. Obstet. Gynecol. Neonatal Nurs. JOGNN.

[3] Laptook A.R., Salhab W., Bhaskar B., Neonatal Research Network (2007). Admission temperature of low birth weight infants: Predictors and associated morbidities. Pediatrics.

[4] Kato S., Iwata O., Iwata S., Yamada T., Tsuda K., Tanaka T., Saitoh S. (2022). Admission temperature of very low birth weight infants and outcomes at three years old. Sci. Rep..

[5] McCall E.M., Alderdice F., Halliday H.L., Vohra S., Johnston L. (2018). Interventions to prevent hypothermia at birth in preterm and/or low birth weight infants. Cochrane Database Syst. Rev..

[6] Hayward J.N., Baker M.A. (1969). A comparative study of the role of the cerebral arterial blood in the regulation of brain temperature in five mammals. Brain Res..

[7] Wang H., Wang B., Normoyle K.P., Jackson K., Spitler K., Sharrock M.F., Miller C.M., Best C., Llano D., Du R. (2014). Brain temperature and its fundamental properties: A review for clinical neuroscientists. Front. Neurosci..

[8] Fox R.H., Solman A.J. (1971). A new technique for monitoring the deep body temperature in man from the intact skin surface. J. Physiol..

[9] Atallah L., Bongers E., Lamichhane B., Bambang-Oetomo S. (2016). Unobtrusive Monitoring of Neonatal Brain Temperature Using a Zero-Heat-Flux Sensor Matrix. IEEE J. Biomed. Health Inform..

[10] Fukaya S., Iwata S., Tsuda K., Hirose A., Kinoshita M., Saitoh S., Iwata O. (2024). Body Size, Cerebral Blood Flow, Ambient Temperature, and Relative Brain Temperatures in Newborn Infants under Incubator Care. Biosensors.

[11] Iwata S., Tachtsidis I., Takashima S., Matsuishi T., Robertson N.J., Iwata O. (2014). Dual role of cerebral blood flow in regional brain temperature control in the healthy newborn infant. Int. J. Dev. Neurosci..

[12] Matsukawa T., Kashimoto S., Ozaki M., Shindo S., Kumazawa T. (1996). Temperatures measured by a deep body thermometer (Coretemp) compared with tissue temperatures measured at various depths using needles placed into the sole of the foot. Eur. J. Anaesthesiol..

[13] Kluckow M., Evans N. (2000). Superior vena cava flow in newborn infants: A novel marker of systemic blood flow. Arch. Dis. Child. Fetal Neonatal Ed..

[14] Dobbing J., Sands J. (1978). Head circumference, biparietal diameter and brain growth in fetal and postnatal life. Early Hum. Dev..

[15] Evans N., Kluckow M., Simmons M., Osborn D. (2002). Which to measure, systemic or organ blood flow? Middle cerebral artery and superior vena cava flow in very preterm infants. Arch. Dis. Child. Fetal Neonatal Ed..

[16] Lutz N.W., Bernard M. (2020). Contactless Thermometry by MRI and MRS: Advanced Methods for Thermotherapy and Biomaterials. iScience.

[17] Copen W.A., Lev M.H., Rapalino O. (2016). Brain perfusion: Computed tomography and magnetic resonance techniques. Handb. Clin. Neurol..

[18] Gautam B., Surak A., Campbell S.M., Kumar M. (2024). Superior Vena Cava Flow in Preterm Infants and Neonatal Outcomes: A Systematic Review. Am. J. Perinatol..

[19] Raichle M.E., Gusnard D.A. (2002). Appraising the brain’s energy budget. Proc. Natl. Acad. Sci. USA.

[20] Kusaka T., Isobe K., Yasuda S., Koyano K., Nakamura S., Nakamura M., Ueno M., Miki T., Itoh S. (2014). Evaluation of cerebral circulation and oxygen metabolism in infants using near-infrared light. Brain Dev..

[21] Zhu M., Ackerman J.J., Yablonskiy D.A. (2009). Body and brain temperature coupling: The critical role of cerebral blood flow. J. Comp. physiology. B Biochem. Syst. Environ. Physiol..

[22] Mulkey S.B., Plessis A.D. (2018). The Critical Role of the Central Autonomic Nervous System in Fetal-Neonatal Transition. Semin. Pediatr. Neurol..

[23] Okken A., Koch J. (1995). Thermoregulation of Sick and Low Birth Weight Neonates.

[24] Thoresen M., Whitelaw A. (2005). Therapeutic hypothermia for hypoxic-ischaemic encephalopathy in the newborn infant. Curr. Opin. Neurol..

[25] Laptook A.R., Corbett R.J. (2002). The effects of temperature on hypoxic-ischemic brain injury. Clin. Perinatol..

[26] Willie C.K., Tzeng Y.C., Fisher J.A., Ainslie P.N. (2014). Integrative regulation of human brain blood flow. J. Physiol..

[27] Vigue B., Ract C., Zlotine N., Leblanc P.E., Samii K., Bissonnette B. (2000). Relationship between intracranial pressure, mild hypothermia and temperature-corrected PaCO_2_ in patients with traumatic brain injury. Intensive Care Med..

[28] Fox P.T., Raichle M.E. (1986). Focal physiological uncoupling of cerebral blood flow and oxidative metabolism during somatosensory stimulation in human subjects. Proc. Natl. Acad. Sci. USA.

[29] Attwell D., Iadecola C. (2002). The neural basis of functional brain imaging signals. Trends Neurosci..

