# Supplementary material for: Investigating Roles of Cerebral Blood Flow to Maintain Thermal Stability of Neonatal Brain Against Cold Stress Using Non-Invasive Probes for Brain Perfusion and Temperature Gradient
Source: Biosensors (Basel). 2026 Feb 20;16(2):127. doi: 10.3390/bios16020127 (PMC12937989; doi:10.3390/bios16020127)
Supplement: Supplementary file 1 [file biosensors-16-00127-s001.zip › biosensors-4099896-supplementary.pdf]

Figure S1. Relationship between Ambient Temperature and Relative Scalp Temperature.

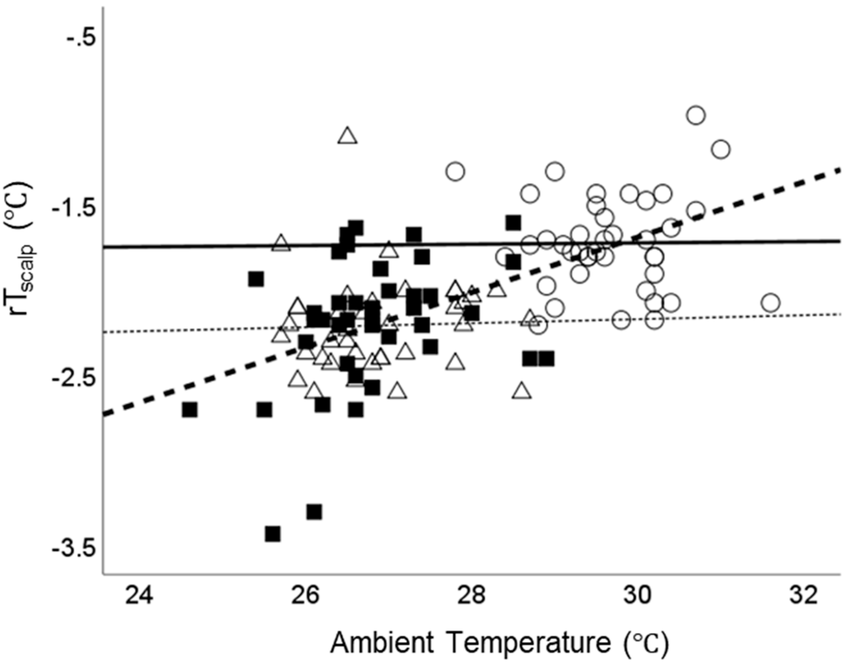

Relationship between ambient temperature and relative scalp temperature (rT_{scalp}) under different ambient conditions. During closed-incubator care (open circles and solid lines), no significant relationship was observed. Immediately after cot transfer (black squares, dashed line), higher ambient temperatures were associated with a higher rT_{scalp}. Two hours after the cot transfer (open triangles, dotted line), no significant relationship was observed.
